# NKG2D-dependent interaction between intestinal epithelial cells and intraepithelial lymphocytes influences food allergic sensitization

**DOI:** 10.1186/2045-7022-3-S3-P46

**Published:** 2013-07-25

**Authors:** M Bol-Schoenmakers, M Marcondes Rezende, L Kruijssen, LL Lanier, L Boon, RH Pieters, JJ Smit

**Affiliations:** 1 Institute for Risk Assessment Sciences, Utrecht University, Utrecht, the Netherlands; 2 Department of Microbiology and Immunology, and the Cancer Research Institute, University of California San Francisco, San Francisco, CA, USA; 3 Bioceros, Utrecht, the Netherlands

## Background

Failure in oral tolerance induction causes allergic sensitization, but the mechanism behind this is still not fully understood. Previously, we found that the number of intraepithelial lymphocytes (IEL, including gammadelta T cells) was decreased during allergic sensitization. In addition, modulation of gammadelta T cells by a specific antibody resulted in enhanced allergic sensitization in a mouse model of peanut allergy. We hypothesized that the response of IEL to epithelial cell (IEC) perturbations due to microbial and non-microbial exposure determines the outcome of the adaptive immune response. The present study aims to investigate the involvement of Natural Killer Group 2 Member D (NKG2D) on IEL and NK cells in allergic sensitization.

## Methods

C57BL/6 mice were treated intragastrically with peanut (PE) plus cholera toxin (CT) and one day later, IEL were isolated and analyzed for activation and NKG2D expression. Furthermore, mice were treated with an anti-NKG2D blocking antibody or an anti-NK1.1 NK cell depleting antibody during intragastric sensitization to PE for 4 weeks using CT as an adjuvant. PE-specific antibodies and T cell responses in the spleen were measured after these 4 weeks.

## Results

Both NKG2D and MHCII expression was upregulated on IEL and gammadelta T cells in Peyer’s patches 24 hr after PE+CT treatment. Furthermore, analysis of the ileum by qPCR showed increased mRNA levels for NKG2D but no significant increase in IL-15 or IL-6. Upon sensitization, the level of PE-specific IgG1 and IgE and PE-induced cytokine production in spleen was significantly increased after blockade of NKG2D. In contrast, depletion of NK cells with anti-NK1.1 did not result in enhanced PE-specific allergic responses after sensitization.

## Conclusion

The present results points towards involvement of NKG2D on cells other than NK cells in allergic sensitization. The response to IEC perturbations due to luminal contents may be mediated by recognition of stress ligands on IEC by the NKG2D receptor on IEL. The present data are indicative of a potential involvement of the interaction between IEL and IEC in allergic sensitization.

## Disclosure of interest

None declared.
